# Impact of the Neck and/or Shoulder Pain on Self-reported Headache Treatment Responses – Results From a Pharmacy-Based Patient Survey

**DOI:** 10.3389/fneur.2022.902020

**Published:** 2022-07-18

**Authors:** Charly Gaul, Heidemarie Gräter, Thomas Weiser, Martin C. Michel, Anette Lampert, Manuel Plomer, Stefanie Förderreuther

**Affiliations:** ^1^Headache Center Frankfurt, Frankfurt am Main, Germany; ^2^Medical Consumer Healthcare, Sanofi-Aventis Deutschland GmbH, Frankfurt am Main, Germany; ^3^Department of Pharmacology, Johannes Gutenberg University, Mainz, Germany; ^4^Department of Neurology, Ludwig Maximilian University, Munich, Germany

**Keywords:** headache, neck and shoulder pain, self-management, ibuprofen, caffeine

## Abstract

Neck and/or shoulder pain (NSP) frequently occurs together with headache. Therefore, we explored how patients with and without concomitant NSP differ in their baseline characteristics and in perceived treatment responses to an analgesic. An anonymous survey was performed among 895 patients with headache (735 self-reported tension-type headache [TTH]) who used an analgesic fixed-dose combination containing 400 mg ibuprofen and 100 mg caffeine as a non-prescription treatment. NSP was abundant among patients in our survey (60%) and was associated with >1 additional day of headache per month. Patients with NSP reported predominantly sedentary work more frequently than those without (40 vs. 29%); they also reported physical tension/poor posture as a perceived trigger factor more frequently (70 vs. 16%). The reported pain reduction was comparable in those with and without concomitant NSP regardless of whether assessed as mean pain rating (from about 6 to 1.5 on a 10-point rating scale), patients experiencing a ≥50% in pain reduction (89.6 vs. 88.8%) or becoming pain-free within 2 h (57 vs. 64%). However, recurrence of pain and use of another dose within the same day were more frequent with than without NSP. We conclude that concomitant NSP is frequent in patients with headache but does not substantially alter responses to a non-prescription medication.

## Introduction

Headache is a widespread phenomenon and affects the life of many people. Previous studies have indicated that headaches may commonly be associated with neck and/or shoulder pain (NSP) (1, 2). A nationwide telephone survey recently conducted in Germany showed that about 51% of the 5,009 respondents suffered from at least one headache attack in the last 12 months preceding the interview (in most cases tension-type headache (TTH) and/or migraine); neck pain was a common accompanying symptom in definite TTH (19%) and migraine (31%) (3). Some data suggest a pathophysiological rationale why headache can be associated with NSP (4).

The German survey (3) showed that only about 22% of women and 17% of men have seen a doctor because of their headache. Headaches were treated with acute pain medication in most of the cases (women: 83%; men: 67%). Usage of ibuprofen (50%), paracetamol (19%), and aspirin (15%) was reported most often (3). Analgesics containing these compounds are available without a prescription (over-the-counter, OTC) in Germany, which is in line with the finding that most patients have not seen a doctor.

OTC analgesics are only available in pharmacies in Germany. Thus, pharmacies are promising touchpoints for contacting and running surveys with headache patients to gather information on their complaints and treatment responses to analgesics. Pharmacy-based patient surveys have provided valuable information in the context of TTH and migraine (5, 6) and other indications that include abdominal spasms and pain (7) or cough and cold (8, 9). To better understand the complaints of headache patients with and without accompanying NSP and their treatment responses, a pharmacy-based survey was performed on patients suffering from headaches that were treated with an OTC analgesic, the fixed-dose combination of 400 mg ibuprofen, and 100 mg caffeine (IbuCaff).

## Patients and Methods

This non-interventional, prospective survey was run in 126 community pharmacies in Germany between February and June 2019. Patients who had purchased a branded IbuCaff product (Thomapyrin TENSION DUO) containing 400 mg ibuprofen and 100 mg caffeine and who consented were handed a questionnaire. This was to be filled out at the participants' own discretion after intake of IbuCaff to treat a pain episode. This questionnaire was to be sent to the institute that collected and analyzed the data (Winicker Norimed GmbH, Nuremburg, Germany), in an envelope provided to the participant. The survey was anonymous, i.e., no data were collected, which allowed for identifying the participants, and participants did not receive any economical support or discount on the product for participating in the study. Applicable German laws and regulations neither require nor recommend the involvement of an ethical committee for an anonymous survey like this. Accordingly, other German studies based on anonymous pharmacy-based patient surveys also did not involve ethical committee approval (5, 8, 9).

Inclusion criteria were purchase and use of the IbuCaff product, willingness and ability to fill out the questionnaire, usage of the product according to the packaging label, and age of ≥18 years. There were no exclusion criteria. The questionnaire contained – among others – questions on demographic variables (age and gender), days of pain within the last 30 days, days with impaired activities due to pain within the last 30 days, type of pain (headache or migraine with or without NSP, and other), analgesics taken in the past for similar pain episodes (prespecified list of OTC analgesics to select previously used treatments for headache), pain intensity of the treated episode (on a numerical pain rating scale (NPRS) with 0 = no pain and 10 = worst pain imaginable), time to onset of pain relief (categories: 0–5, 6–15, 16–30, 31–45, 46–60, and >60 min), assessments of time to onset of pain relief (categories: very fast, fast, moderately fast, and slow), pain 2 h after intake of IbuCaff, intake of another tablet, recurrence of pain within the same day, efficacy, tolerability (categories: very good, good, not so good, and bad), and willingness to buy the product again and to recommend it to others. In case of handwritten notes, these were checked for adverse events (AEs). According to the recommendations by the International Headache Society, one important piece of information on the effects of headache treatments is the fraction of patients who become pain-free 2 h after treatment administration, as well as the fraction experiencing partial pain relief after 2 h (10, 11); therefore, this has also been assessed. All efficacy analyses are based solely on participants indicating headache or migraine as the primary reason for the use of IbuCaff, whereas those indicating “other” were only included in the tolerability analyses.

### Data Analysis

Data management and analysis were performed using SAS, version 9.2 (by Winicker Norimed GmbH) based on a statistical analysis plan developed by the authors and using GraphPad Prism, version 9.3.1 (by the authors). Data are reported as means ± SD and/or as medians with interquartile ranges (IQR) for continuous and as a percentage for categorical variables. In line with the exploratory character of the study, no hypothesis-testing statistical analysis was performed as recommended by various guidelines on data robustness (12, 13). All reported *p*-values for group-wise comparisons were descriptive only and were done with unpaired *t-*tests (age, days with pain or impaired daily life per month, and pain intensity), paired *t*-tests (pre- vs. post-pain intensity), Mann-Whitney tests (onset of pain relief, global efficacy, and tolerability rating), or contingency analyses (gender, sedentary work, trigger factors, number of patients with pain relief, pain-free patients, recurrence of pain, and use of second analgesic dose), and depending on the type of data. A preprint of a previous version of the manuscript has been made available at https://www.preprints.org/manuscript/202011.0631/v1. Based on the suggestion of a referee, additional *post-hoc* analyses were performed to explore the effects of concomitant NSP within the group of headache patients with and without self-reported migraine (see Supplementary Material).

## Results

**Figure 1:**
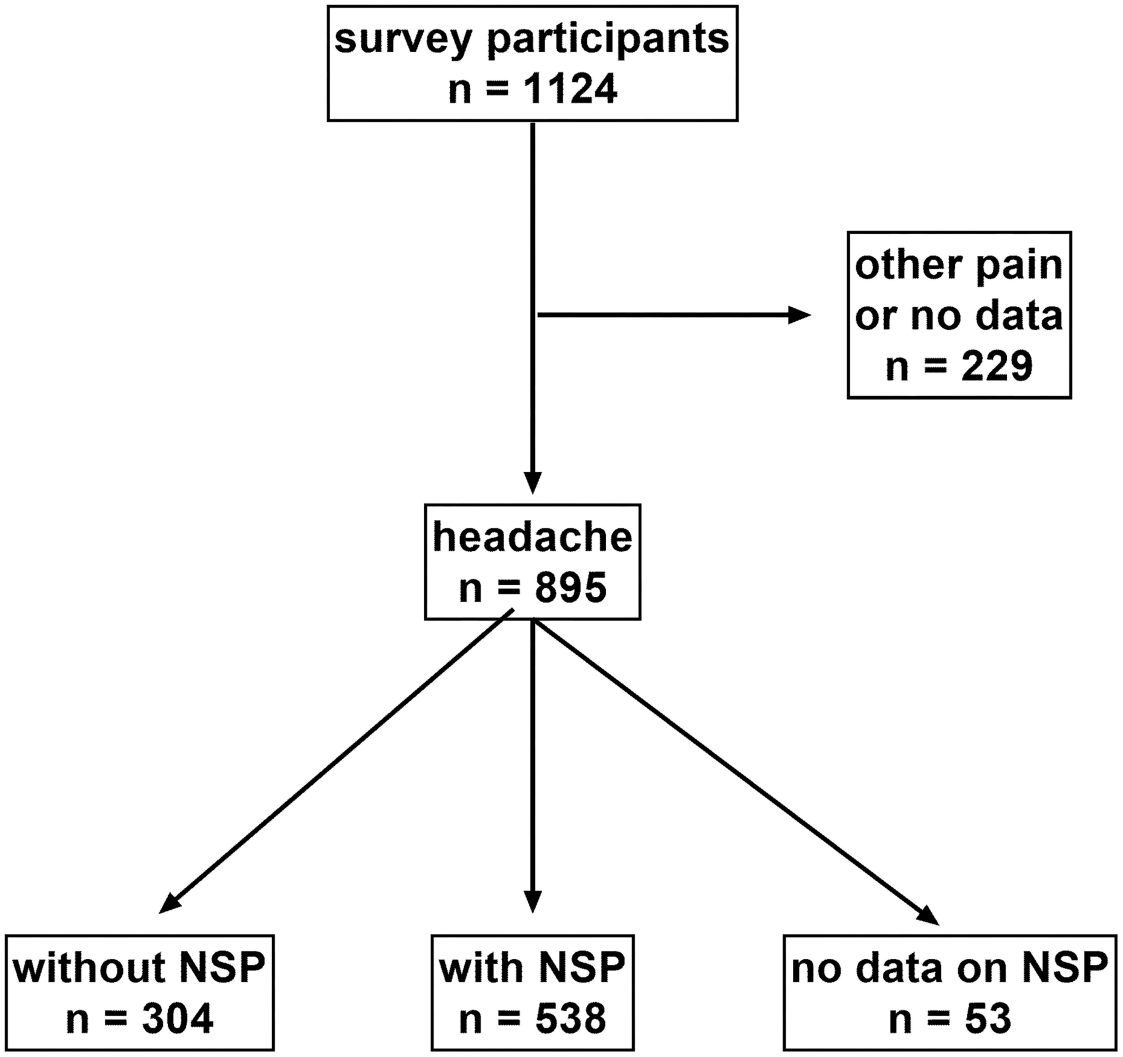
STROBE diagram on the disposition of patients.

Among 1,124 participants fulfilling the inclusion criteria and providing analyzable questionnaires, 895 reported using IbuCaff for the treatment of headache and 110 for pain other than headache; no information on the type of pain was given in 119 questionnaires (Figure 1). Since no formal headache diagnosis was performed, data for self-reported “headache” (*n* = 735) and “migraine” (*n* = 160) were pooled and constituted the efficacy population. In total, 304 participants suffered from headache without NSP and 538 from headache with NSP. All 1,124 subjects constituted the safety population.

Participants suffering from headache with or without NSP had comparable age and gender distributions (Table 1). Those treating headache with NSP more often reported mainly sedentary work (40 vs. 29% without NSP), more headache days per month (5.7 vs. 4.3), more days with impaired daily life per month (1.7 vs. 0.9), and on average reported greater pain intensity (6.3 vs. 5.8 points).

**Table 1:** Baseline characteristics of headache patients.

	**Headache without NSP**	**Headache with NSP**	***p*-value**
Age (years, mean ± SD)	41.1 ± 14.3	41.9 ± 13.9	0.4407
Gender (% female)	68	70	0.5867
Predominantly sedentary work (%)	29	40	0.0379
Days with pain per month (mean ± SD)	4.3 ± 3.7	5.7 ± 4.6	<0.0001
Days with impaired daily life per month (mean ± SD)	0.9 ± 1.8	1.7 ± 3.2	0.0002
Pain intensity (mean ± SD)	5.8 ± 1.8	6.3 ± 1.8	0.0012

When asked for the perceived pain triggers, a higher fraction of patients with headache plus NSP reported tension/poor posture as the main trigger of their pain (70 vs. 18%; Table 2). Conversely, all other perceived triggers were reported more often by headache sufferers without NSP.

**Table 2:** Perceived triggers for the assessed headache attack.

**Pain triggers**	**Headache without NSP**	**Headache with NSP**	***p*-value**
Stress	125 (41%)	198 (37%)	0.2378
(Physical) tension/poor posture	49 (16%)	377 (70%)	<0.0001
Nutrition (e.g., dehydration)	31 (10%)	46 (9%)	0.4559
Weather sensitivity	88 (29%)	82 (15%)	<0.0001
Common cold / other diseases	31 (10%)	28 (5%)	0.0076
Hormonal disbalance (e.g., due to menstruation)	26 (9%)	39 (7%)	0.5037
Other	18 (6%)	15 (3%)	0.0275
Do not know	28 (9%)	16 (3%)	0.0002

*Multiple mentions were allowed*.

On average, the participants mentioned 1.9 different medications in their headache history that included about half having used ibuprofen (free acid or lysine salt; Table 3).

**Table 3:** Analgesics taken in the participants' headache history.

	**Without NSP**	**With NSP**
**Analgesics taken in the past**		
Ibuprofen	161 (28%)	291 (29%)
Ibuprofen lysinate	101 (17%)	209 (21%)
Aspirin	66 (11%)	113 (11%)
Paracetamol	93 (16%)	165 (16%)
Naproxen	75 (13%)	30 (3%)
Other combination than IbuCaff	76 (13%)	113 (11%)
Other analgesic	41 (7%)	44 (4%)
No analgesic	11 (2%)	7 (1%)

*Multiple mentions were allowed*.

**Figure 2:**
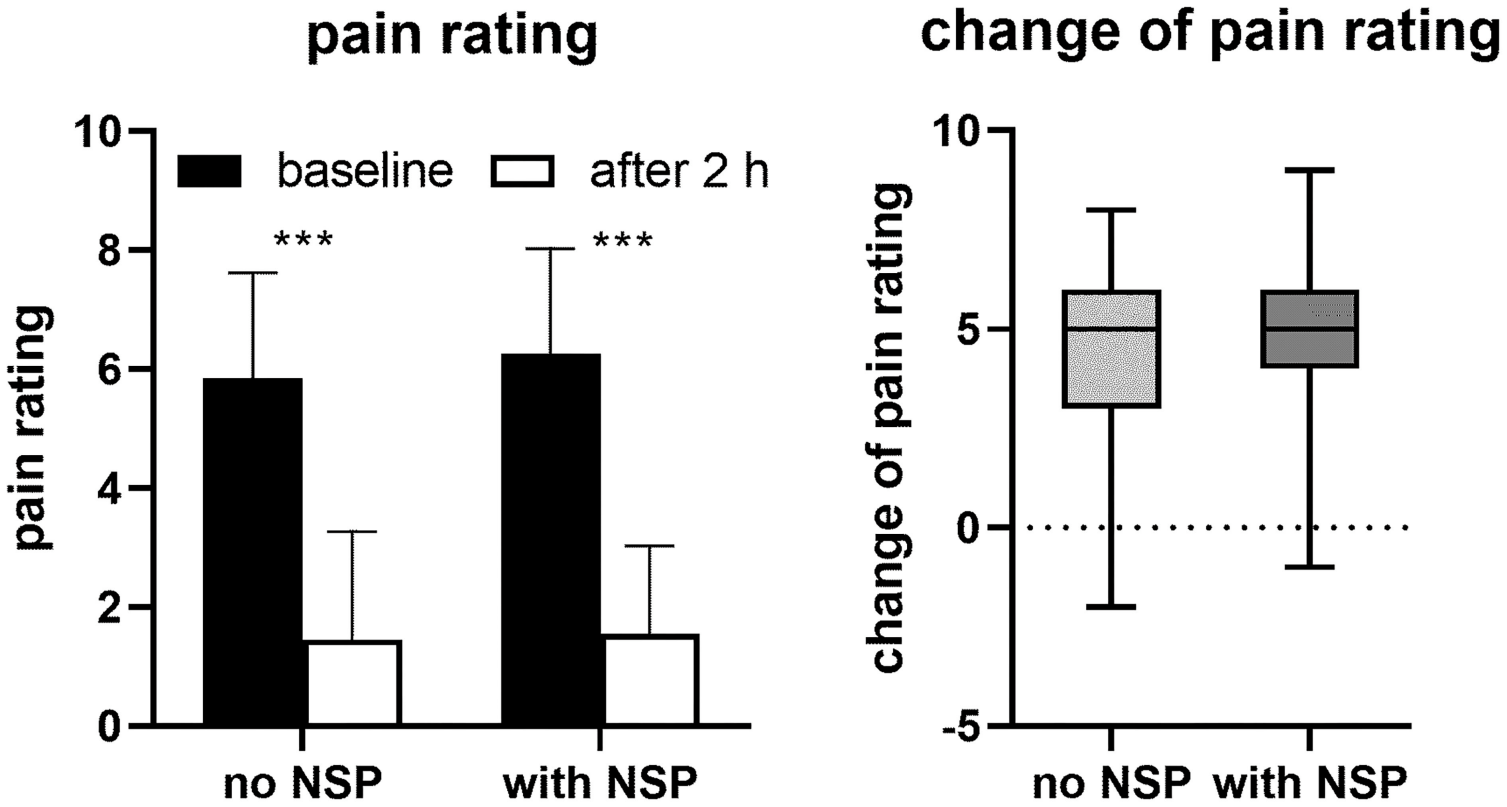
Pain ratings (means ± SD) reported by participants before and 2 h after intake of IbuCaff (**left panel**) and box and whisker plot of intra-individual changes. ***: *p* < 0.0001 based on paired *t-*tests.

Mean baseline headache pain was greater with NSP than without (see Table 1) but was similarly reduced 2 h after intake of IbuCaff pain in both groups (to 1.5 ± 1.5 points with and to 1.5 ± 1.8 points without NSP, Figure 2), indicating comparable efficacy irrespective of the absence or presence of NSP. Similarly, comparable numbers of patients with or without NSP reported pain relief 2 h after intake of IbuCaff (90% with and 89% without NSP, *p* = 0.7288; defined reduction of pain by ≥50%) or to become pain-free (57 and 64%, *p* = 0.0676; defined as 0 or 1 point on the NPRS). However, recurrence of pain within the same day after the intake of one tablet of IbuCaff was reported by 39% without and 55% with NSP (*p* < 0.0001). Accordingly, more patients with NSP took another dose of IbuCaff than those without (53 vs. 36%, *p* < 0.0001). Although mean pain reduction after 2 h and percentages of patients experiencing pain relief were similar, more headache sufferers with NSP (91%, when compared to 84% without NSP) would recommend IbuCaff to others and were willing to buy it again (93 vs. 84%). The effects of IbuCaff were also similar in the absence and presence of NSP if subgroups of patients with and without self-reported migraine were considered, except that the percentage of patients reporting to be pain-free after 2 h was lower in subjects with migraine (Supplementary Material).

**Figure 3:**
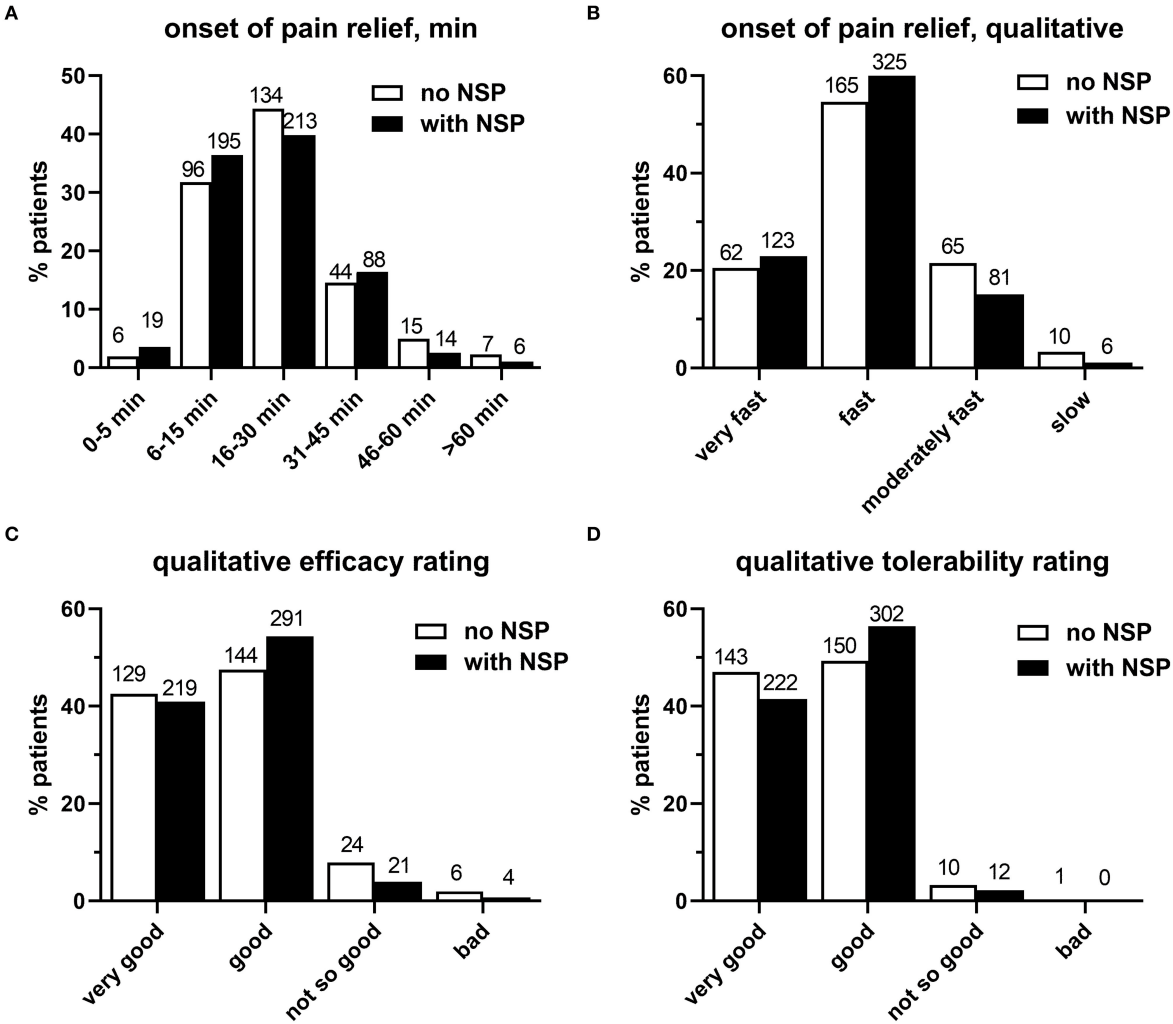
**(A)** Onset of pain relief. **(B)** Patient ratings of speed for the perceived onset of pain relief. **(C)** Patient ratings for IbuCaff efficacy. **(D)** Ratings for IbuCaff tolerability. The numbers of patients for the different groups are shown on the top of columns.

The onset of pain relief after intake of the first dose of IbuCaff was similarly reported to occur mostly within 6–15 or 16–30 min in both groups (Figure 3A, *p* = 0.6234). This was also applied to the assessment of the perceived speed of onset of pain relief, which was largely rated as very fast or fast (Figure 3B, *p* = 0.6857).

Perceived efficacy did not differ relevantly between the two groups and with top ratings (very good/good) in about 90% of subjects (Figure 3C, *p* = 0.8857). Similarly, perceived tolerability was rated as very good or good by >90% of the 842 survey participants in both groups (Figure 3D, *p* = 0.6857). All patients (i.e., including those who treated pain other than headache) gave similar results (very good/good/not so good/ bad: 43/54/3/0.1%). Specific AEs based on handwritten notes include 1 case each of trembling, heart racing, stomach pain, dizziness, and worsened neck pain. One participant reported in the handwritten notes to have taken IbuCaff for the indication of fever, which is an off-label use.

## Discussion

Headache is often accompanied by other complaints, such as neck or back pain (1–3). Using a group of self-reported headache or migraine sufferers, our data confirm the frequent comorbidity between headache and NSP. While baseline symptoms differ between those with and without concomitant NSP, the efficacy of IbuCaff appears to be comparable in subjects with and without concomitant NSP.

### Limitations

Non-interventional/observational pharmacoepidemiology studies can provide useful information on the effects of treatments in general practice. A recent review pointed out that “[…] following robust assessment of efficacy by R[andomized] C[ontrolled] T[rial]s, O[bservational] P[harmacoepidemiology] studies is able to assess whether an intervention is effective in day-to-day clinical practice, which often includes more heterogeneous patient groups and less precise diagnostic criteria than might feature in a randomized controlled trial (RCT)” (14). Pharmacy-based patient surveys are a form of the non-interventional study that is frequently used to study patients and their treatment responses in indications often managed by self-care using OTC medications, such as headache (5, 6) and other indications (7–9). Such non-interventional studies cannot prove efficacy because they lack a control group. However, the efficacy of ibuprofen for the treatment of headache and the enhancement of analgesic effects by caffeine are undisputed. However, caffeine's role is somewhat complex, since the cessation of caffeine intake (higher doses for prolonged time) can cause withdrawal headache, and caffeine intake is discussed as a risk factor for chronification of migraine (15, 16) although this appears to set in only with 5 or more cups of coffee per day (17), a much larger amount than what is contained in 1–2 tablets of the IbuCaff preparation. Moreover, controlled, randomized trials have demonstrated the superiority of IbuCaff relative to monotherapy or placebo (18, 19). The magnitude of a potential placebo-effect contribution to the patient-reported findings cannot be determined from the present data. Another important difference between observational studies and RCTs is that knowledge of receiving active treatment improves perceived efficacy as also shown in patients with migraine (20). Therefore, it was not surprising that reported efficacy in the present study appeared higher than in previous studies with classic analgesics in patients with headache. Thus, the present findings should not be misinterpreted as proof of efficacy; however, they provide an impression of what patients and healthcare professionals can realistically expect in a real-life setting; they should be informative regarding the impact of concomitant NSP on baseline symptoms and treatment responses.

Another limitation results from relying on self-diagnosis of NSP and headache and, within headache, whether this was migraine or not. Previous research had shown that a large fraction of patients can adequately self-diagnose their type of headache, but some cannot (5). Therefore, initially, the role of NSP in headache patients with and without migraine was not explored, but added based on a suggestion within the peer-review process of this manuscript; these data should be interpreted with caution. As the vast majority of patients with headache do not see a doctor about this and apply self-management (3), relying on self-diagnosis appears inevitable if the study population is desired to be representative of headache sufferers.

Finally, questionnaires directed at patients must use patient-friendly language and should be kept short to ensure maximum participation by the users. For this reason, the questionnaire-mentioned symptoms and triggers in layperson language, e.g., awkward position, but did not provide formal definitions of what these terms mean. This raises the possibility that some participants misinterpreted some terms, which creates a potential limitation for the interpretation of their responses. However, we feel that this is a necessary and useful compromise in the setting of a pharmacy-based patient survey.

### Findings at Baseline

About 64% of the survey participants suffering from headache also reported NSP. Subjects with and without concomitant NSP were similar in age and gender distribution. However, a greater fraction of participants with NSP reported to mainly work in sedentary positions and had more severe pain on more days per month, with less quality of life. Thus, NSP can be considered an aggravating co-factor for the burden of headache.

Not surprisingly, perceived (physical) tension/poor posture was rated as the most frequent trigger of headache and most strongly differentiated subjects with NSP from those without. Other perceived triggers were reported more often by subjects not suffering from NSP, suggesting that tension/poor posture exceeds the importance of other factors in headache sufferers with NSP.

In line with the treatments reported by a larger sample of German headache sufferers (3), patients had mainly treated their complaints in the past with ibuprofen (acid or lysinate), paracetamol, and/or aspirin, with no remarkable differences between subjects with or without NSP.

### Perceived Treatment Effects

The addition of caffeine to analgesics has been recommended for enhanced pain relief (16) and is generally considered to be safe in doses used for this purpose (21) and not to cause dependence (15). There is no evidence that medication overuse headache occurs more frequently with the addition of caffeine than with analgesic monotherapy. IbuCaff is an analgesic product, which was introduced into the German market in 2018. This fixed-dose combination has shown superior efficacy in an acute dental pain model as compared to ibuprofen, caffeine, and placebo (19), and a slightly different combination was superior to ibuprofen alone in TTH (22). Since ibuprofen alone has been shown to effectively suppress TTH (23) and migraine (24), it is not surprising that IbuCaff relieved pain also in our survey. Mean pain reduction 2 h after intake, as well as the percentages of patients becoming pain-free or experienced pain relief (≥50% pain reduction), was similar in those suffering from headache with or without NSP: Around 60% became pain-free 2 h after intake, and about 90% reported pain relief. Thus, in the real-world setting of our study, most patients were benefitted from the treatment with IbuCaff. In line with the stronger complaints in those suffering from headache with NSP, higher percentages of these patients reported the headache to recur within the same day and took another pill of IbuCaff. We consider this to be expected because patients suffering more intrinsically are more difficult to treat.

The onset of pain relief was reported within 15 min in 34–38% and within 30 min in the vast majority of patients (about 78%). This corresponded well with the assessment of the onset of action, which was perceived by 75% (without NSP) and 84% (with NSP) as “very fast” or “fast.” Similar results were reported in a pharmacy-based survey on another caffeine-containing fixed-dose combination (aspirin, paracetamol, and caffeine) (5).

In general, satisfaction with IbuCaff onset of action, efficacy, and tolerability was rather high. Only very few AEs were reported, and about 97% of participants reported tolerability to be “very good” or “good.” This corresponds well with data from a RCT, where 91% of IbuCaff-treated patients reported tolerability to be “excellent,” “very good,” or “good” [(19) and data on file]. We find it interesting that more headache sufferers with NSP were willing to buy it again despite mean pain reduction after 2 h and percentages of patients experiencing pain relief being similar.

## Conclusions and Outlook

In conclusion, the present data confirm the frequent comorbidity between headache and migraine and NSP. They expand our knowledge by demonstrating that concomitant NSP is associated with a worse headache but also that the efficacy of IbuCaff in the treatment of headache is largely maintained in those with NSP. This investigation adds useful information on the perceived effects in patients treating headache with or without NSP in a day-to-day setting. However, several questions remain open that need addressing in future studies. This includes studies in which the type of headache has been confirmed by a healthcare professional and studies on chronic headache. Moreover, it remains to be investigated whether the maintained therapeutic efficacy of IbuCaff in headache/migraine with NSP can be extrapolated to headache/migraine treatments that are not expected to have direct effects on NSP, such as triptans. A third relevant task would be to better understand how headache and NSP are related mechanistically, for instance, the interplay between nociceptive afferents and sensitization of trigeminocervical neurons (25). Finally, the impact of concomitant NSP on the development of medication-overuse-headache needs to be explored (26).

## Data Availability Statement

The raw data supporting the conclusions of this article will be made available by the authors, without undue reservation.

## Ethics Statement

Ethical review and approval was not required for the study on human participants in accordance with the local legislation and institutional requirements. The patients/participants provided their written informed consent to participate in this study.

## Author Contributions

CG supported the preparation of the survey, analyzed, and interpreted the data and read, commented, and approved the manuscript. HG was involved in the conceptualization of the work, implemented the project, such as collection, analysis, and interpretation of the data, and revised and approved the manuscript. TW was involved in the conceptualization of the work, the analysis and interpretation of the data, contributed to the manuscript concept, and revised and approved the manuscript. MM developed the statistical analysis plan and drafted the manuscript. AL was involved in the conceptualization of the work, interpretation of the data, and revised and approved the manuscript. MP was involved in the conceptualization of the work, contributed to the primary manuscript concept, and had the overall supervision of the project. SF supported the preparation of the survey, analyzed, and interpreted the data and read, commented, and approved the manuscript. All authors have contributed to the manuscript draft for critical intellectual content and read and approved the final manuscript version.

## Funding

The underlying survey had been funded by Sanofi-Aventis Germany GmbH.

## Conflict of Interest

CG has received honoraria for consulting and lectures within the past 3 years from Allergan, Eli Lilly, Grünenthal, Hormosan, Lundbeck Perfood, Novartis Pharma, Sanofi-Aventis, TEVA, and Weber & Weber; he is honorary secretary of the German Migraine and Headache Society. HG, TW, AL, and MP are employees of Sanofi-Aventis Deutschland GmbH. MM is a consultant for Sanofi-Aventis Deutschland GmbH. SF received consultant, speaker, and/or advisory board member honoraria from Eli Lilly, Hormosan, Lundbeck, Novartis, Sanofi-Aventis, and Teva.

## Publisher's Note

All claims expressed in this article are solely those of the authors and do not necessarily represent those of their affiliated organizations, or those of the publisher, the editors and the reviewers. Any product that may be evaluated in this article, or claim that may be made by its manufacturer, is not guaranteed or endorsed by the publisher.
