# Supplementary material for: Impact of the Neck and/or Shoulder Pain on Self-reported Headache Treatment Responses – Results From a Pharmacy-Based Patient Survey
Source: Front Neurol. 2022 Jul 18;13:902020. doi: 10.3389/fneur.2022.902020 (PMC9339896; doi:10.3389/fneur.2022.902020)

Online Supplement to

Impact of neck and/or should pain on self-reported headache treatment responses – results from a pharmacy-based patient survey

Charly Gaul[1], Heidemarie Gräter[2], Thomas Weiser[2], Martin C. Michel[3*], Anette Lampert[2], Manuel Plomer[2], Stefanie Förderreuther[4]

This Online Supplement provides analyses similar to those described in the main manuscript but now stratified by subgroups of self-diagnosed headache vs. migraine. While the non-migraine group is likely to include headache forms other than tension-type headache (TTH) including unrecognized migraine, it is also likely that it consists predominantly of TTH patients. For ease of reading, this online supplement refers to it as TTH, but this is not intended to indicate that a self-diagnosis of TTH vs. other forms of non-migraine headache had been made; rather it is intended to avoid confusion when the term "headache" in the main paper refers to all study participants. Due to the post-hoc exploratory character of these subgroup comparisons, no p-values were calculated.

Supplemental Table 1: Demographics and baseline data according to type of headache in participants with and without concomitant NSP. Complaint data refer to last 30 days prior to purchase of medication. Data are means ± SD for continuous variables (medians shown additionally in parentheses for some variables) and percentages for categorical variables. 43 subjects with TTH and 10 with migraine did not report on the presence or absence of NSP and were not included here.

	**TTH**		**Migraine**	
	**Without NSP**	**With NSP**	**Without NSP**	**With NSP**
N	245	447	59	91
Age, years	40.7 ± 14.6	42.4 ± 14.1	42.7 ± 13.4	39.2 ± 12.3
Gender, % female	64.2	67.6	81.4	79.1
Mainly sitting activity, %	29.6	38.3	27.6	47.3
Days with headache per month	4.1 ± 3.8 (3)	5.6 ± 4.7 (4)	5.2 ± 3.1 (5)	6.5 ± 4.0 (6)
Days with impaired daily life per month	0.7 ± 1.7 (0)	1.6 ± 3.3 (0)	1.7 ± 2.3 (1)	2.3 ± 3.0 (1)
Pain intensity, 0-10 NPRS	5.5 ± 1.7	6.0 ± 1.8	7.2 ± 1.6	7.4 ± 1.3

Supplemental Table 2: Perceived triggers for the assessed headache attack in participants with and without concomitant NSP. Data are shown as % of responders, multiple mentions were allowed.

	**TTH**		**Migraine**	
	**Without NSP**	**With NSP**	**Without NSP**	**With NSP**
Stress	39	36	49	37
(Physical) tension/poor posture	15	74	20	52
Nutrition (e.g., dehydration)	12	7	3	15
Weather sensitivity	27	12	39	30
Common cold / other diseases	12	5	3	4
Hormonal disbalance (e.g., due to menstruation)	7	6	14	12
Other	6	3	7	1
Do not know	10	3	3	1

<u>Supplemental Figure 1:</u> Change of pain ratings, % of patients with a reduction by ≥50% and % of patient reporting to be pain-free 2 h after intake of IbuCaff. The data on pain reduction are means ± SD (upper panel) whereas those on % of patients with a reduction by ≥50% and % of patient reporting to be pain-free 2 h after intake of IbuCaff are counts.

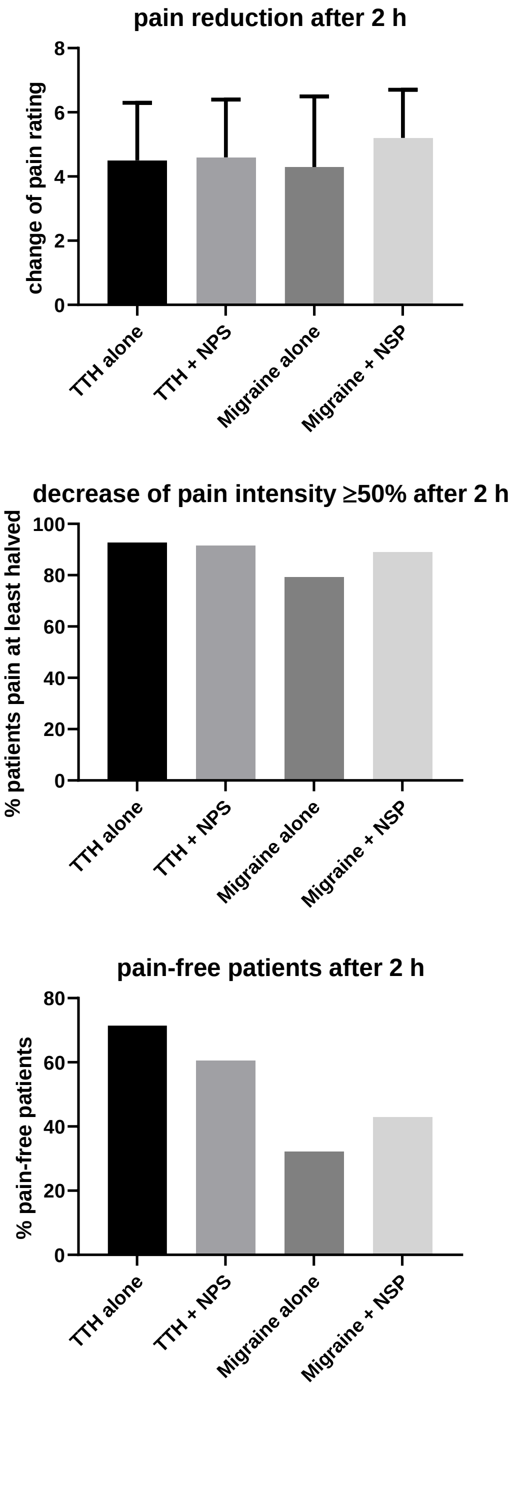

Supplement: Supplementary file 1 [file Data_Sheet_1.PDF]
